# Coarse limestone does not alleviate the negative effect of a low Ca/P ratio diet on characteristics of tibia strength and growth performance in broilers

**DOI:** 10.1016/j.psj.2020.06.037

**Published:** 2020-07-03

**Authors:** Y.X. Hu, P. Bikker, M. Duijster, W.H. Hendriks, J. van Baal, M.M. van Krimpen

**Affiliations:** ∗ Wageningen University & Research, Wageningen Livestock Research, 6700 AH Wageningen, The Netherlands; † Wageningen University & Research, Animal Nutrition Group, 6700 AH Wageningen, The Netherlands; ‡ R&D Department, De Heus Animal Nutrition B.V., 6710, BJ Ede, The Netherlands

**Keywords:** particle size of limestone, Ca/P ratio, phosphorus and calcium digestibility, broilers

## Abstract

The hypothesis was tested that an increased digestion of coarse compared with fine limestone can alleviate the negative effects of a low dietary Ca/P ratio on the growth performance and characteristics of tibia strength (**CTS**) in broilers. A total of 1,152 Ross 308 broiler chickens received a standard commercial starter feed from day 0 to 13. From day 14 onward, birds received 1 of 12 diets containing 1 of 6 Ca/P ratios (0.50, 0.75, 1.00, 1.25, 1.50, and 1.75) and 1 of 2 limestone particle sizes (<500 [fine] and 500 to 2,000 [coarse] μm) in a study with a 6 × 2 factorial arrangement of treatments. Total P content was fixed at 5.5 g/kg for all treatment diets. Each treatment was replicated 6 times with 16 birds per replicate pen. On day 20 and 21, twelve birds per pen were randomly selected from 4 of the 6 replicate pens for tibia analysis and digesta collection from different gut segments. The apparent Ca digestibility was higher for fine than coarse limestone in the jejunum (*P* = 0.043). However, this difference in Ca digestibility disappeared for the low, whereas it remained for the high Ca/P ratios in the proximal (*P*_interaction_ = 0.067) and distal (*P*_interaction_ = 0.052) ileum. In addition, coarse limestone improved apparent P digestibility in the proximal and distal ileum (*P* < 0.001) but not in the jejunum (*P* = 0.305). Regardless of limestone particle size, reducing dietary Ca/P ratio linearly improved apparent Ca and P digestibility in the proximal and distal ileum (*P* < 0.001). Moreover, decreasing dietary Ca/P ratio linearly (*P* < 0.001) and quadratically (*P* < 0.046) reduced the CTS. Reducing dietary Ca/P ratio linearly (*P* < 0.003) and quadratically (*P* ≤ 0.006) decreased body weight gain and increased feed conversion ratio. For both fine and coarse limestone, the optimal Ca/P ratio was 1.00 to 1.25 to optimize apparent Ca and P digestibility while maintaining growth performance and CTS. Reducing Ca/P ratio from 1.75 to 1.00 improved distal ileal Ca and P apparent digestibility from 36.6 to 53.7% and 48.0 to 58.3%, respectively. In conclusion, coarse limestone is equally digestible with fine limestone at a low Ca/P ratio but is less digestible at a high Ca/P ratio, and the optimal Ca/P ratio in the diet is 1.00 to 1.25 for both fine and coarse limestone.

## Introduction

Phosphorus (**P**) and calcium (**Ca**) are essential macronutrients for all animals and play an important role in numerous physiological processes including bone formation (Shao et al., 2019). Improving P utilization helps to alleviate the global mineral P depletion, save feed costs as well as reduce environmental pollution of P. Calcium plays a pivotal role in P utilization as it hampers P absorption via Ca–P complexation in the gut, but it is essential to bind P to form hydroxyapatite in bone (Misiura et al., 2018). Reducing the dietary Ca/P ratio is an effective way to improve P digestibility (Rodehutscord, 2016), but overreduction of the dietary Ca/P ratio compromises growth performance and characteristics of tibia strength (**CTS**, Van Krimpen et al., 2013).

Compared with fine limestone (particle size < 500 μm), coarse limestone (particle size 1,000–2,000 μm) has been shown to be more digestible in broilers, with a true ileal digestibility of 70 vs. 40% (Anwar et al., 2016, 2017). It, therefore, seems possible to reduce the dietary Ca/P ratio through the use of coarse limestone while maintaining growth performance and bone development. In addition, in the presence of microbial phytase, ileal P digestibility was reduced by pulverized limestone (particle size < 75 μm), whereas it was not affected by the particulate limestone (particle size 402 μm) (Kim et al., 2018). The P digestion seems to be related to the particle size of limestone, and the coarser limestone may have a less negative impact on P digestion. It is hypothesized that coarse limestone with its higher digestibility in broilers can alleviate the negative effect of a low Ca/P ratio on growth performance and bone development and may improve P digestibility compared with fine limestone. The objective of the present study was to determine the impact of limestone particle size and dietary Ca/P ratio on apparent Ca and P digestibility in different gut segments, CTS, as well as growth performance in broilers.

## Materials and methods

The experiment was conducted in the broiler research accommodation of De Heus (Eerde, the Netherlands). All procedures complied with the Dutch law on animal experiments, and the study was approved by the Ethical Committee of Wageningen University & Research, the Netherlands (no. 2016.D-0065.004).

### Animal Housing and Management

A total of 1,152 zero-day-old Ross 308 male broilers housed in 72 pens (1 m^2^, 16 birds per pen) on wood shavings (0.9–1.0 kg/m^2^) were used. The barn was mechanically ventilated, and the temperature was controlled by a climate computer. The room temperature was set at 35°C on the day of arrival and thereafter gradually decreased by 1°C per day to 20°C to 18°C. A light: dark schedule of 18:6 was used in the barn with a light intensity of 20 lux. Continuous lighting (24L:0D) was used on the first 2 D of the experiment (day 0–1), as well as the 4 D before and during dissection (day 18–21). Dead birds or birds with visible malfunction (e.g., scissor beak, ascites, and torticollis) were removed and weighed at the time of removal. Birds had *ad libitum* access to water and feed throughout the experiment (day 0–39). All birds received a normal commercial starter feed from day 0–13 (2,973 kcal/kg ME, 212 g/kg CP, 8.6 g/kg Ca, 5.5 g/kg P). Experimental diets were provided to the animals from day 14 onward.

### Experimental Treatments and Diets

On day 14, birds were weighed and randomly allotted to 1 of 12 treatments in a 6 × 2 factorial arrangements of treatments including 6 dietary Ca/P ratios (0.50, 0.75, 1.00, 1.25, 1.50, and 1.75) and 2 limestone particle sizes (<500 [fine] and 500–2,000 μm [coarse]). Each treatment was replicated 6 times. The coarse and fine limestone were obtained from the same limestone product (Sibelco, Maastricht, the Netherlands) via sieving through a 500 μm screen (Retsch GmbH, Haan, Germany).

A basal diet was made and then split into 12 equal portions, to which the required amount of coarse or fine limestone was added at the expense of diamol (Damolin, Kønsborgvej, Denmark) according to the experimental design. The basal diet met or exceeded the minimum requirement of all nutrients except Ca (CVB, 2016) and included titanium dioxide at 5 g/kg as an indigestible marker. The composition of the grower (day 14–29) and finisher (day 30–39) diets is shown in Table 1 and Supplementary Table 1, respectively. The P content was 5.5 and 5.1 g/kg in all grower and finisher diets, respectively. The Ca content was 2.7 to 9.6 g/kg for the grower and 2.5 to 8.8 g/kg for the finisher diets. The Ca content was below the minimum requirement of 7.0 and 6.0 g/kg for grower and finisher birds, respectively (Ca/P ratio 1.25, CVB, 2016) in the 3 low Ca/P ratios while exceeding the requirement in the 2 high Ca/P ratios. The experimental diets were made in increasing order of Ca/P ratio. Experimental diets were produced by a feed production plant for research diets (Research Diet Services in Wijk bij Duurstede, The Netherlands) using a double mixing procedure to assure equal composition of the experimental diets. All experimental diets were given in pelleted form to prevent segregation.

**Table 1:** Composition and nutrient content of grower diets with fine or coarse limestone and incremental Ca/P ratios (g/kg as-fed basis, day 14 to 29).

Item	Particle size limestone											
	Fine^1^						Coarse^2^					
Ca/P ratio	0.50	0.75	1.00	1.25	1.50	1.75	0.50	0.75	1.00	1.25	1.50	1.75
Ingredients												
Corn	354	354	354	354	354	354	354	354	354	354	354	354
Wheat	300	300	300	300	300	300	300	300	300	300	300	300
Soybean meal, extracted	255	255	255	255	255	255	255	255	255	255	255	255
Soybean oil	36.5	36.5	36.5	36.5	36.5	36.5	36.5	36.5	36.5	36.5	36.5	36.5
Rapeseed meal, extracted	10.1	10.1	10.1	10.1	10.1	10.1	10.1	10.1	10.1	10.1	10.1	10.1
Monosodium phosphate	7.0	7.0	7.0	7.0	7.0	7.0	7.0	7.0	7.0	7.0	7.0	7.0
Monocalcium phosphate	2.5	2.5	2.5	2.5	2.5	2.5	2.5	2.5	2.5	2.5	2.5	2.5
Limestone (fine)^1^	3.6	7.2	10.8	14.4	18.0	21.6	0.0	0.0	0.0	0.0	0.0	0.0
Limestone (coarse)^2^	0.0	0.0	0.0	0.0	0.0	0.0	3.6	7.2	10.8	14.4	18.0	21.6
Salt	0.3	0.3	0.3	0.3	0.3	0.3	0.3	0.3	0.3	0.3	0.3	0.3
L-Val (98%)	0.9	0.9	0.9	0.9	0.9	0.9	0.9	0.9	0.9	0.9	0.9	0.9
Met (99%)	2.5	2.5	2.5	2.5	2.5	2.5	2.5	2.5	2.5	2.5	2.5	2.5
L-Lys (79%)	2.5	2.5	2.5	2.5	2.5	2.5	2.5	2.5	2.5	2.5	2.5	2.5
Thr (88%)	1.1	1.1	1.1	1.1	1.1	1.1	1.1	1.1	1.1	1.1	1.1	1.1
Salinocox^3^	0.6	0.6	0.6	0.6	0.6	0.6	0.6	0.6	0.6	0.6	0.6	0.6
Diamol^4^	18.1	14.5	10.9	7.3	3.7	0.0	18.1	14.5	10.9	7.3	3.7	0.0
Titanium dioxide (TiO_2_)	5.0	5.0	5.0	5.0	5.0	5.0	5.0	5.0	5.0	5.0	5.0	5.0
Premix^5^	5.0	5.0	5.0	5.0	5.0	5.0	5.0	5.0	5.0	5.0	5.0	5.0
Calculated nutrients												
Dry matter	878	878	878	878	878	878	878	878	878	878	878	878
ME, kcal/kg	3,029	3,029	3,029	3,029	3,029	3,029	3,029	3,029	3,029	3,029	3,029	3,029
Crude protein	194	194	194	194	194	194	194	194	194	194	194	194
Lys	1.02	1.02	1.02	1.02	1.02	1.02	1.02	1.02	1.02	1.02	1.02	1.02
Met	0.49	0.49	0.49	0.49	0.49	0.49	0.49	0.49	0.49	0.49	0.49	0.49
Met + Cys	8.6	8.6	8.6	8.6	8.6	8.6	8.6	8.6	8.6	8.6	8.6	8.6
Thr	8.0	8.0	8.0	8.0	8.0	8.0	8.0	8.0	8.0	8.0	8.0	8.0
Ca	2.7	4.1	5.5	6.9	8.2	9.6	2.7	4.1	5.5	6.9	8.2	9.6
Total P (P)	5.5	5.5	5.5	5.5	5.5	5.5	5.5	5.5	5.5	5.5	5.5	5.5
Available P (aP)	3.2	3.2	3.2	3.2	3.2	3.2	3.2	3.2	3.2	3.2	3.2	3.2
Ca/P	0.50	0.75	1.00	1.25	1.50	1.75	0.50	0.75	1.00	1.25	1.50	1.75
Analysed nutrients												
Dry matter	888	895	896	895	896	891	891	898	898	896	895	890
Crude protein	187	191	188	187	189	192	188	189	186	190	189	192
Crude fat	60.7	61.4	59.8	59.4	60.1	60.0	60.6	59.9	60.7	61.3	61.2	59.6
Ca	3.1	4.4	6.3	7.2	9.3	10.5	3.1	4.6	5.8	7.8	8.7	10.1
P	5.8	5.8	5.8	5.8	5.9	5.9	5.8	5.9	5.9	5.9	5.9	5.8
Ca/P	0.54	0.76	1.08	1.23	1.59	1.78	0.53	0.78	0.99	1.32	1.47	1.73

1 Analyzed Ca content: 41.1%. Particle size distribution: > 2,000 μm, 0.0%; 1,000–2,000 μm, 0.0%; 500–1,000 μm, 0.2%; 250–500 μm; 33.6%; <250 μm, 66.2%. Geometric mean diameter 160 μm, geometric standard deviation 96 μm.

2 Analyzed Ca content: 41.1%. Particle size distribution: >2,000 μm, 0.0%; 1,000–2,000 μm, 59.3%; 500–1,000 μm, 40.4%; 250–500 μm, 0.1%; <250 μm, 0.2%. Geometric mean diameter 1,062 μm, geometric standard deviation 387 μm.

3 Sacox, Antwerp, Belgium.

4 Damolin, Kønsborgvej, Denmark.

5 Provided per kg of diet: 12,000 IE retinol, 2,400 IE cholecalciferol, 50 mg dl-a-tocopherol, 1.5 mg menadione, 2.0 mg thiamine, 7.5 mg riboflavin, 3.5 mg pyridoxine, 20 mg cyanocobalamins, 35 mg niacin, 12 mg D-pantothenic acid, 460 mg choline chloride, 1.0 mg folic acid, 0.2 mg biotin, 80 mg iron, 12 mg copper, 85 mg manganese, 60 mg zinc, 0.4 mg cobalt, 0.8 mg iodine, 0.1 mg selenium, 125 mg anti-oxidant mixture.

### Sample Collection

Feed samples were collected in the feed mill during production. Birds were weighed on day of arrival (day 0), experimental diet allotment (day 14), dissection and sample collection (day 20–21), the end of the grower period (day 29), and the end of the experiment (day 39). On day 20 and 21, twelve birds per pen were randomly selected from 4 of the 6 replicate pens per treatment and euthanized by electrocution before blood was collected from the carotid artery of 2 dissected birds per pen. A number of 4 replicate pens per treatment was considered adequate because the focus was not on comparison of individual treatments but on determination of linear and quadratic effects of Ca/P ratio and the interactions, based on 6 treatment groups, including 24 pens. Serum was harvested after centrifuging at 3,000 g × 10 min at 4°C. After exsanguination, the chest cavity and the abdomen were opened. The gastrointestinal tract (**GIT**) was ligated by tie wraps into 8 segments including crop, proventriculus plus gizzard, duodenum, jejunum, the first half of ileum (proximal ileum), the second half of ileum (distal ileum), ceca, and colon to prevent digesta flowing between gut compartments. The proventriculus and gizzard were not separated but merged as 1 GIT segment because the proventriculus did not contain any substantial amount of digesta. The jejunum was defined as the GIT segment from the end of the duodenal loop to the Meckel's diverticulum. The ileum was divided into 2 equal parts to determine the apparent prececal or distal ileal digestibility of P and Ca (Rodehutscord, 2013). After ligating, the GIT was removed from the birds. The digesta were quantitatively collected per segment by flushing with deionized water. The ceca were emptied by gently squeezing because of the viscosity of the cecal digesta. The digesta samples were pooled per segment per pen, and the pH was determined using a pH meter (Seven2Go, Mettler Toledo, Schwerzenbach, Switzerland). Because of the interference with urine excretion, Ca and P digestibility in the colon was not measured. All digesta samples were stored in the freezer (−20°C) until further analysis. The right tibia was removed from 6 of the 12 dissected birds per pen and stored (−20°C) before a bone breaking test. The remaining birds were kept until day 39 to determine the effect of dietary treatments on growth performance in the overall experimental period from day 14 to 39.

### Observations and Chemical Analysis

The particle size distribution of the pelleted diets was determined using wet sieving, and the geometric mean diameter (**GMD**) and geometric standard deviation (**GSD**) were calculated (ASAE, 2008). To examine the contrast of limestone particle size in pelleted diets, the 4 diets of the lowest and highest Ca/P ratio for both fine and coarse limestone were incinerated at 550°C. Particle size distribution of the feed ash was subsequently determined using dry sieving. Diets were also analyzed for dry matter by drying at 103°C (ISO, 6496), N was analyzed using the Kjeldahl method, and crude protein content was calculated as N × 6.25 (FOSS, Hillerod, Denmark; ISO, 5983). Particle size distribution of the coarse and fine limestone was determined using dry sieve analysis. The digesta samples were freeze-dried and ground to pass a 1-mm sieve using a Retsch ZM 100 mill (Retsch GmbH, Germany). The ground digesta and feeds were incinerated at 550°C (ISO, 5984), P content was determined spectrophotometrically (Evolution 201; Thermo scientific, Waltham, MA; ISO, 6491), and Ca was determined using atomic absorption spectrometry (AA240 FS; Varian, CA; ISO, 6869). Titanium content was determined using a spectrophotometer (Evolution 201; Thermo Scientific) after destruction by H_2_SO_4_ (FOSS), according to Myers et al. (2004). Serum Ca and P were analyzed using a Cobas 8000 modular analyzer for clinical analysis with C701 Photometric measuring unit (Roche Diagnostics Limited, Rotkreuz, Switzerland). Commercial available kits were used to analyze the serum alkaline phosphatase (ALP, Diatools AG, Villmergen, Switzerland), 1,25-dihydroxycholecalciferol (1,25(OH)_2_D_3_, Immunodiagnostic Systems GmbH, Germany) and parathyroid hormone (Immunotopics, San Clemente, CA). The CTS was determined by a bone breaking test using an Instron Texture Analyzer (type 5,564, MA). Energy to fracture, maximum compressive load, stiffness, and diameter were determined as characteristics of tibia strength, as described by Guz et al. (2019).

Solubility of P was determined in the freeze-dried digesta of the crop, gizzard, and jejunum of the lowest and highest Ca/P ratio diet for both coarse and fine limestone. The dried digesta were incubated in a buffer solution to mimic in vivo digesta conditions (pH and dry matter) as determined in the subsequent gut segment. This was done to determine the amount of potentially digestible P entering to the next gut segment. Specifically, 1 g of the ground crop digesta was incubated in 2 mL buffer solution to mimic in vivo gizzard digesta conditions (pH 3.2, DM 33%), and 1 g of ground gizzard or jejunum sample was incubated in 6 mL buffer solution to mimic in vivo jejunum/ileum digesta condition (pH 5.8, DM 14%). The soluble P was extracted by mixing an aliquot of digesta on a horizontal shaker at 150 rpm and 42°C for 30 (crop digesta) or 40 (gizzard and jejunum digesta) min. Thereafter, sample were centrifuged at 3,000 g for 15 min, supernatant with soluble P was discarded, the residue with insoluble was dried, and the P content was subsequently determined.

### Calculations and Statistical Analysis

The following equation was used to calculate the nutrient digestibility in gut segments:

$$\text{Digestibility coefficient},\;\%=\left(1-\frac{{\text{X}}_{\text{digesta}}}{{\text{X}}_{\text{diet}}}\times \frac{{\text{Ti}}_{\text{diet}}}{{\text{Ti}}_{\text{digesta}}}\right)\times 100$$

where X_digesta_ and Ti_digesta_ are the nutrient (Ca or P) and Ti content in the freeze-dried digesta (g/kg), respectively and X_diet_ and Ti_diet_ are the nutrient (Ca or P) and Ti content in the diet (g/kg), respectively.

The digesta mean retention time (**MRT**) in different gut segments was calculated according to de Vries and Gerrits (2018) as:

$$\text{MRT},\text{min}=\frac{1,440\times {\text{Ti}}_{\text{digesta}}\times {\text{W}}_{\text{digesta}}}{\text{FI}\times {\text{Ti}}_{\text{diet}}}$$

where 1,440 are the minutes per day (min/day), Ti_digesta_ the Ti content in the freeze-dried digesta (g/kg), W_digesta_ the quantitative weight of the dried digesta (kg) in each of the respective segments, FI the feed intake over 24 h (kg/day), and Ti_diet_ the Ti content in the diet (g/kg).

The P solubility was calculated using the equation:

$$\text{P solubility},\%=\frac{{\text{W}}_{\text{digesta}}\times {\text{P}}_{\text{digesta}}-{\text{W}}_{\text{residue}}\times {\text{P}}_{\text{residue}}}{{\text{W}}_{\text{digesta}}\times {\text{P}}_{\text{digesta}}}\times 100$$

where W_digesta_ is the weight of freeze-dried digesta used for this test (g), P_digesta_ is the P content in the freeze-dried digesta (g/kg), W_residue_ is the dried weight of digesta residue after discarding the supernatant (g), and P_residue_ is the P content in the dried digesta residue (g/kg).

The CTS was determined on individual tibia (6 tibias per pen), but for all other measurements, samples were pooled per pen and determined at a pen level. Pen was the experimental unit for data analysis. All data except the CTS were subjected to a 2-way ANOVA using the GLM procedure of SAS 9.4 (SAS Institute, Cary, NC). The limestone particle size, Ca/P ratio, and their interaction were used as fixed effects. The CTS were subjected to a 2-way ANOVA using the MIXED procedure with limestone particle size, Ca/P ratio, and their interaction as fixed effects and pen as random effect. The distribution, variance, and homogeneity of studentized residuals were visually checked via graphics plotted using the ODS GRAPHICS function. The LSMEANS procedure with a PDIFF option was used to estimate the difference between means if a significant interaction was obtained. A CONTRAST procedure was used to estimate the linear and quadratic effect of dietary Ca/P ratio regardless of the limestone particle size. The coefficients for the CONTRAST procedure were obtained using the IML procedure. Probability was considered significant at *P* ≤ 0.05 and a trend at 0.05 < *P* < 0.1.

## Results

The analyzed Ca and P contents in the diets were slightly higher than the calculated levels, but the analyzed Ca/P ratios were in good agreement with the designed ratios (Table 1). The GMD and GSD for the coarse limestone were 1,062 and 387 μm, respectively, and 160 and 96 μm for the fine limestone. As for the diets, wet sieve analysis indicated that the GMD and GSD were increased with the incremental Ca/P ratio for the coarse limestone and were approximately 174 (163–183) and 261 (243–280) μm for the 6 diets, respectively. In the ash fraction of the lowest and highest Ca/P ratio diets, more coarse particles (>500 μm) were observed for coarse than fine limestone diets. Accordingly, the GMD of feed ashes was greater for coarse than the fine limestone (116 vs. 101 at a Ca/P ratio of 0.50 and 196 vs. 116 at a Ca/P ratio of 1.75; Table 2). Seventy-six percent of the coarse limestone particles included in the diet were recovered in the feed ashes.

**Table 2:** Dry sieve analysis of ashes of pelleted diets with the highest (1.75) and lowest (0.50) Ca/P ratio for both fine and coarse limestone, % unless otherwise specified.^1^

Particle size	Fine		Coarse	
Ca/P ratio	0.50	1.75	0.50	1.75
Sieve diameter, μm				
>2,500	0.07	0.88	0.24	0.23
1,250–2,500	0.07	0.67	0.59	2.60
1,000–1,250	0.07	0.82	1.42	6.29
630–1,000	0.34	0.72	2.43	18.9
320–630	1.28	3.92	2.08	4.85
160–320	6.40	12.6	5.63	5.89
63–160	78.8	64.2	80.8	49.1
<63	13.0	16.1	6.82	12.1
GMD, μm	101	116	116	196
GSD, μm	41	85	73	255

Abbreviations: GMD, geometric mean diameter; GSD, geometric standard deviation.

1 The Ca content and particle size distribution of the coarse and fine limestone are provided in Table 1.

During the whole experimental period, the birds realized a high feed intake and growth rate, and their average body weight at the end of the experiment met or exceeded the performance objectives of the breeding company (ROSS, 2014).

### Ca and P Apparent Digestibility and Solubility

No significant interaction was observed between limestone particle size and Ca/P ratio on P or Ca apparent digestibility for any of the gut segments (Table 3). For Ca apparent digestibility, a trend for an interactive effect was observed in the proximal (*P*_interaction_ = 0.067) and distal (*P*_interaction_ = 0.052) ileum. The Ca apparent digestibility was not different between the fine and coarse limestone at the low Ca/P ratio, whereas it was higher for the fine than the coarse limestone at the high Ca/P ratio. In the jejunum, the Ca apparent digestibility was higher for the fine than for the coarse limestone (*P* = 0.043) irrespective of the Ca/P ratio. Regardless of limestone particle size, the incremental Ca/P ratio linearly (*P* < 0.001) reduced Ca apparent digestibility in the proximal and distal ileum but not in the jejunum (*P* = 0.246). The P apparent digestibility was higher for the treatments with coarse limestone (*P* < 0.001) in the proximal and distal ileum, but not in the jejunum (*P* = 0.305). Regardless of limestone particle size, increasing the Ca/P ratio, linearly (*P* < 0.001) and quadratically (*P* < 0.001) decreased P apparent digestibility in the proximal and distal ileum. The incremental Ca/P ratio also linearly (*P* < 0.001) decreased and tended to quadratically (*P* = 0.088) decrease P apparent digestibility in the jejunum. The P solubility was not affected by the limestone particle size, Ca/P ratio, or their interactions in the crop, gizzard, or jejunum (Table 4).

**Table 3:** Effect of dietary Ca/P ratio and particle size of limestone on P and Ca apparent digestibility in the jejunum and ileum in broilers^1^^,^^2^^,^^3^, %.

Particle size	Ca/P ratio	P Digestibility			Ca digestibility		
		Jejunum	Prox. Ileum	Distal ileum	Jejunum	Prox. ileum	Distal ileum
Fine	0.50	61.8	72.6	71.8	49.9	59.0	61.9
	0.75	55.6	63.4	65.0	45.5	55.1	57.2
	1.00	49.3	55.4	55.2	49.8	52.2	54.1
	1.25	45.4	50.2	51.9	47.1	42.3	42.9
	1.50	45.6	50.3	50.9	46.2	44.7	49.9
	1.75	40.6	46.8	47.5	39.0	41.5	42.7
Coarse	0.50	62.7	75.5	75.8	48.0	61.6	66.0
	0.75	55.4	66.6	67.8	35.9	58.9	60.9
	1.00	53.8	60.5	61.3	43.0	52.4	53.3
	1.25	48.0	54.4	56.8	42.7	37.1	45.4
	1.50	43.6	51.4	53.0	33.1	42.5	39.1
	1.75	45.0	50.2	48.4	42.0	29.4	30.5
Pooled SEM		2.84	1.36	1.67	4.51	2.74	3.27
Particle size mean							
Fine		49.7	56.4	57.0	46.2	49.2	51.5
Coarse		51.4	59.8	60.5	40.8	47.0	49.2
Pooled SEM		1.16	0.56	0.68	1.84	1.12	1.34
Ca/P ratio mean							
	0.50	62.3	74.1	73.8	49.0	60.3	64.0
	0.75	55.5	65.0	66.4	40.7	57.0	59.1
	1.00	51.6	58.0	58.3	46.4	52.3	53.7
	1.25	46.7	52.3	54.4	44.9	39.7	44.2
	1.50	44.6	50.9	52.0	39.7	43.6	44.5
	1.75	42.8	48.5	48.0	40.5	35.5	36.6
Pooled SEM		2.00	0.96	1.18	3.19	1.94	2.31
*P*-value							
Particle size		0.305	<0.001	<0.001	0.043	0.178	0.250
Ca/P ratio		<0.001	<0.001	<0.001	0.246	<0.001	<0.001
Particle size × Ca/P ratio		0.832	0.785	0.673	0.556	0.067	0.052
Linear (Ca/P ratio)		<0.001	<0.001	<0.001	0.089	<0.001	<0.001
Quadratic (Ca/P ratio)		0.088	<0.001	0.001	0.951	0.568	0.718

1 Data are presented as treatment means, 4 replicate pens per treatment (n = 4).

2 Prox. ileum = proximal ileum, the first half of the ileum.

3 Ca content and particle size distribution of the fine and coarse limestone are shown in Table 1.

**Table 4:** Effect of Ca/P ratio and particle size of limestone on P solubility in freeze dried digesta, solubilized under the conditions (pH and dry matter content) of the digesta in the subsequent segment of the digestive tract in broilers.^1^^,^^2^

Particle size	Ca/P ratio	Crop	Gizzard	Jejunum
Fine	0.50	30.0	56.5	80.0
	1.75	31.1	55.7	72.8
Coarse	0.50	26.3	73.2	67.1
	1.75	27.7	56.1	78.3
Pooled SEM		3.63	5.88	6.26
*P*-value				
Particle size		0.386	0.172	0.569
Ca/P ratio		0.750	0.153	0.760
Particle size × Ca/P ratio		0.965	0.192	0.166

1 Data are presented as treatment means, 4 replicate pens per treatment (n = 4).

2 The Ca content and particle distribution of the coarse and fine limestone are shown in Table 1.

### Tibia and Serum Characteristics

No interaction was observed between particle size of the limestone and Ca/P ratio on CTS (Table 5) or serum characteristics (Table 6). Limestone particle size did not affect CTS or serum characteristics either. Regardless of limestone particle size, tibia maximum compressive load, fracture energy, and stiffness were linearly (*P* < 0.001) and quadratically (*P* < 0.046) increased with an incremental Ca/P ratio, whereas tibia diameter was not affected. Increasing dietary Ca/P ratio linearly (*P* < 0.001) increased serum Ca content and tended to linearly decrease (*P* = 0.073) serum 1,25-(OH)_2_D_3_ content. The incremental Ca/P ratio linearly (*P* < 0.001) decreased serum P content. However, the Ca/P ratio had no impact on the serum ALP or parathyroid hormone.

**Table 5:** Effect of dietary Ca/P ratio and particle size of limestone on characteristics of tibia strength in broilers determined by a bone breaking test as described by Guz et al. (2019).^1^^,^^2^

Particle size	Ca/P ratio	Maximum compressive load, N	Energy to fracture, N·mm	Stiffness, N/mm	Thickness, mm
Fine	0.50	139	185	105	5.44
	0.75	179	206	138	5.57
	1.00	216	264	145	5.69
	1.25	203	233	156	5.42
	1.50	235	274	181	5.73
	1.75	212	244	171	5.60
Coarse	0.50	144	189	106	5.50
	0.75	154	181	126	5.42
	1.00	216	263	160	5.73
	1.25	210	240	168	5.66
	1.50	203	216	162	5.76
	1.75	224	249	163	5.59
Pooled SEM		13.0	20.7	10.4	0.111
Particle size mean					
Fine		197	234	149	5.58
Coarse		192	223	148	5.61
Pooled SEM		5.31	8.45	4.26	0.045
Ca/P ratio mean					
	0.50	142	187	106	5.47
	0.75	167	194	132	5.50
	1.00	216	264	153	5.71
	1.25	207	237	162	5.54
	1.50	219	245	172	5.75
	1.75	218	247	167	5.60
Pooled SEM		9.23	14.7	7.39	0.078
*P*-value					
Particle size		0.459	0.345	0.722	0.610
Ca/P ratio		<0.001	0.001	<0.001	0.066
Particle size × Ca/P ratio		0.413	0.567	0.522	0.663
Linear (Ca/P ratio)		<0.001	0.001	<0.001	0.063
Quadratic (Ca/P ratio)		0.001	0.046	0.004	0.201

1 Data are presented as treatment means, 6 tibias per replicate pen and 4 replicate pens per treatment (n = 24).

2 The Ca content and particle distribution of the coarse and fine limestone are shown in Table 1.

**Table 6:** Effect of dietary Ca/P ratio and particle size of limestone on serum characteristics in broilers.^1^^,^^2^^,^^3^

Particle size	Ca/P ratio	Ca, mmol/L	P, mmol/L	ALP, U/L	PTH, pg/mL	1,25-Vit D_3,_ pg/mL
Fine	0.50	2.28	2.53	6,293	210	228
	0.75	2.63	2.50	6,040	121	202
	1.00	2.43	2.08	5,737	105	218
	1.25	2.53	2.03	6,247	198	201
	1.50	2.60	2.18	6,766	48	188
	1.75	2.83	1.80	6,053	206	173
Coarse	0.50	2.30	2.63	5,664	246	229
	0.75	2.58	2.55	5,895	91	199
	1.00	2.53	2.30	4,492	184	210
	1.25	2.58	2.28	5,595	91	170
	1.50	2.43	2.15	6,139	238	252
	1.75	2.70	1.83	7,157	188	180
Pooled SEM		0.097	0.148	823	55.0	21.0
Particle size mean						
Fine		2.55	2.19	6,189	148	202
Coarse		2.52	2.29	5,824	173	207
Pooled SEM		0.040	0.060	336	23.1	8.57
Ca/P ratio mean						
	0.50	2.29	2.58	5,979	228	229
	0.75	2.61	2.53	5,968	106	201
	1.00	2.48	2.19	5,115	145	214
	1.25	2.56	2.16	5,921	145	186
	1.50	2.52	2.17	6,453	143	220
	1.75	2.77	1.82	6,605	197	177
Pooled SEM		0.069	0.105	582	38.9	14.8
*P*-value						
Particle size		0.607	0.231	0.447	0.449	0.692
Ca/P ratio		0.001	<0.001	0.554	0.302	0.124
Particle size × Ca/P ratio		0.702	0.920	0.794	0.186	0.368
Linear (Ca/P ratio)		0.001	<0.001	0.276	0.903	0.073
Quadratic (Ca/P ratio)		0.953	0.918	0.242	0.056	0.950

1 Data are presented as treatment means, 4 replicate pens per treatment (n = 4).

2 ALP = alkaline phosphatase; PTH = parathyroid hormone; 1, 25 vit-D3 = 1,25-dihydroxycholecalciferol.

3 The Ca content and particle distribution of the coarse and fine limestone are shown in Table 1.

### Growth Performance

Limestone particle size did not impact growth performance, except that coarse limestone reduced feed conversion ratio (**FCR**) from day 14 to 20 (*P* = 0.010, Table 7). Overall, incremental dietary Ca/P ratio improved body weight gain from day 14 to 20, and this positive effect of dietary Ca/P ratio on body weight gain was also observed in the total grower (day 14–29) and overall experimental period (day 14–39, Supplementary Table 2). From day 14 to 20 to day 14 to 29, the FCR was linearly (*P* < 0.001) and quadratically (*P* < 0.004) reduced by incremental dietary Ca/P ratio. However, this effect of dietary Ca/P ratio on FCR disappeared in the finisher (day 29–39) and overall (day 14–39) period. The feed intake was linearly (day 14–29, *P* = 0.041) or quadratically (day 14–20, *P* = 0.045) increased by incremental Ca/P ratio in the grower period and continued to be increased by dietary Ca/P ratio in the finisher and overall experimental period.

**Table 7:** Effect of Ca/P ratio and particle size of limestone on growth performance in the grower period in broilers.^1^^,^^2^^,^^3^^,^^4^

Particle size	Ca/P ratio	Day 14 to 20			Day 14 to 29		
		BWG, g	FI, g	FCR, g/g	BWG, g	FI, g	FCR, g/g
Fine	0.50	462^b,c^	664	1.44	1,271	1,824	1.43
	0.75	482^a,b^	678	1.41	1,429	2,020	1.41
	1.00	480^a,b^	657	1.37	1,397	1,921	1.38
	1.25	485^a^	662	1.36	1,444	1,999	1.38
	1.50	490^a^	673	1.37	1,489	2,049	1.38
	1.75	457^c^	631	1.38	1,383	1,919	1.39
Coarse	0.50	444^c^	638	1.44	1,237	1,800	1.46
	0.75	478^a,b^	662	1.39	1,382	1,949	1.41
	1.00	490^a^	671	1.37	1,399	1,943	1.39
	1.25	489^a^	659	1.35	1,414	1,948	1.38
	1.50	487^a^	665	1.37	1,384	1,912	1.38
	1.75	493^a^	667	1.35	1,471	2,015	1.37
Pooled SEM		7.4	10.2	0.008	39.1	64.8	0.01
Particle size mean							
Fine		476	661	1.39	1,402	1,955	1.40
Coarse		478	661	1.38	1,381	1,928	1.40
Pooled SEM		3.1	4.2	0.003	15.9	26.5	0.01
Ca/P ratio mean							
	0.50	453	651	1.44	1,254	1,812	1.45
	0.75	480	670	1.40	1,406	1,985	1.41
	1.00	485	665	1.37	1,398	1,932	1.39
	1.25	487	661	1.36	1,429	1,974	1.38
	1.50	488	669	1.37	1,437	1,981	1.38
	1.75	475	649	1.37	1,427	1,967	1.38
Pooled SEM		5.2	7.2	0.006	27.6	45.8	0.01
*P*-value							
Particle size		0.346	0.937	0.010	0.391	0.476	0.901
Ca/P ratio		<0.001	0.201	<0.001	<0.001	0.081	<0.001
Particle size × Ca/P ratio		0.020	0.055	0.404	0.302	0.601	0.738
Linear (Ca/P ratio)		0.003	0.779	<0.001	<0.001	0.041	<0.001
Quadratic (Ca/P ratio)		<0.001	0.045	<0.001	0.006	0.107	0.004

1 Data are presented as treatment means, 6 replicate pens per treatment (n = 6).

2 BWG = body weight gain; FI = feed intake; FCR = feed conversion ratio.

3 The Ca content and particle distribution of the coarse and fine limestone are shown in Table 1.

4 Each treatment had an equal number of 6 pens with 16 birds per pen before day 20, and an unequal number of 4 pens with 4 birds per pen and 2 pens with 16 birds per pen after day 21.

### Digesta pH and MRT

No interaction was found between particle size of the limestone and Ca/P ratio on digesta pH in gut segments except for the duodenum (*P*_interaction_ = 0.026, Table 8). No other effects of limestone particle size on digesta pH was observed in either of the segments, except in the ceca (*P* = 0.026), where digesta pH was higher for the fine than the coarse limestone. Increasing dietary Ca/P ratio linearly increased (*P* < 0.011) digesta pH in the proventriculus plus gizzard, ceca, and colon, whereas it linearly decreased (*P* = 0.002) digesta pH in the crop. Digesta pH was not affected by the Ca/P ratio in jejunum, proximal, or distal ileum.

**Table 8:** Effect of Ca/P ratio and particle size of limestone on digesta pH in different intestinal segments in broilers.^1^^,^^2^^,^^3^

Particle size	Ca/P ratio	Crop	Prov. + gizzard	Duodenum	Jejunum	Prox. Ileum	Distal ileum	Ceca	Colon
Fine	0.50	5.51	3.09	5.95^a,b,c^	5.77	5.94	5.71	6.07	5.70
	0.75	5.21	2.96	5.89^a,b,c^	5.73	5.74	5.67	6.06	5.60
	1.00	5.01	3.03	5.89^a,b,c^	5.73	5.93	6.14	6.17	5.83
	1.25	4.93	3.53	5.74^c^	5.73	5.69	6.42	6.55	5.83
	1.50	5.06	3.52	5.94^a,b,c^	5.81	6.00	6.15	6.59	6.12
	1.75	4.80	3.52	6.05^a^	5.79	5.88	6.49	6.69	6.41
Coarse	0.50	5.32	3.07	5.78^c^	5.76	5.78	5.66	5.96	5.55
	0.75	4.99	2.91	5.95^a,b,c^	5.75	5.95	5.82	5.96	5.70
	1.00	5.06	3.15	5.89^a,b,c^	5.75	5.99	6.20	5.99	6.03
	1.25	5.08	3.15	5.99^a,b^	5.77	6.18	6.07	6.34	6.14
	1.50	5.02	3.48	5.84^b,c^	5.78	6.11	6.17	6.35	6.22
	1.75	5.00	3.39	5.82^b,c^	5.74	5.76	5.94	6.34	5.98
Pooled SEM		0.135	0.098	0.073	0.044	0.150	0.249	0.148	0.121
Particle size mean									
Fine		5.09	3.28	5.91	5.76	5.86	6.10	6.36	5.92
Coarse		5.08	3.19	5.88	5.76	5.96	5.98	6.16	5.94
Pooled SEM		0.057	0.040	0.030	0.018	0.061	0.102	0.060	0.051
Ca/P ratio mean									
	0.50	5.42	3.08	5.87	5.77	5.86	5.69	6.02	5.63
	0.75	5.10	2.94	5.92	5.74	5.85	5.75	6.01	5.65
	1.00	5.04	3.09	5.89	5.74	5.96	6.17	6.08	5.93
	1.25	5.01	3.34	5.87	5.75	5.94	6.25	6.45	5.99
	1.50	5.04	3.50	5.89	5.80	6.06	6.16	6.47	6.17
	1.75	4.90	3.46	5.94	5.77	5.82	6.22	6.52	6.20
Pooled SEM		0.096	0.069	0.051	0.031	0.107	0.176	0.105	0.085
*P*-value									
Particle size		0.889	0.142	0.455	0.922	0.268	0.415	0.026	0.812
Ca/P ratio		0.035	<0.001	0.884	0.803	0.637	0.098	0.001	<0.001
Particle size × Ca/P ratio		0.573	0.238	0.026	0.918	0.305	0.711	0.960	0.052
Linear (Ca/P ratio)		0.002	<0.001	0.526	0.528	0.647	0.011	<0.001	<0.001
Quadratic (Ca/P ratio)		0.179	0.428	0.722	0.539	0.272	0.212	0.921	0.643

1 Data are presented as treatment means, 4 replicate pens per treatment (n = 4).

2 Prov. + gizzard = proventriculus plus gizzard; Prox. ileum = proximal ileum, the first half of ileum.

3 The Ca content and particle distribution of the coarse and fine limestone are shown in Table 1.

No interaction was found between particle size of the limestone and Ca/P ratio on the digesta MRT (Table 9). Limestone particle size did not affect digesta MRT in the gut segments, except that MRT was greater for the coarse limestone in the proventriculus plus gizzard (*P* = 0.004) and ceca (*P* = 0.034). Increasing Ca/P ratio linearly increased (*P* = 0.004) the digesta MRT in the crop, but it linearly decreased (*P* < 0.001) MRT in the proventriculus plus gizzard and ceca. The MRT was not affected by the Ca/P ratio in the duodenum, jejunum, or ileum.

**Table 9:** Effect of Ca/P ratio and particle size of limestone on MRT in different intestinal segments in broilers^1^^,^^2^^,^^3^, min.

Particle size	Ca/P ratio	Crop	Prov. + gizzard	Duodenum	Jejunum	Prox. Ileum	Distal ileum	Ceca
Fine	0.50	15.6	30.1	1.36	43.6	39.0	44.9	2.41
	0.75	23.7	26.2	2.04	45.8	37.3	49.6	1.47
	1.00	24.5	23.0	1.26	44.8	39.3	43.9	1.31
	1.25	28.3	20.5	1.64	50.1	46.7	47.9	0.87
	1.50	30.6	22.1	1.22	43.2	41.9	50.4	0.81
	1.75	32.9	23.8	1.26	43.6	40.2	48.0	0.60
Coarse	0.50	20.6	30.1	1.70	48.9	39.0	52.1	2.32
	0.75	27.3	32.7	1.26	47.1	39.5	52.7	2.44
	1.00	34.9	34.2	1.18	45.7	38.9	53.9	1.82
	1.25	27.6	28.6	1.02	46.4	38.1	44.0	1.23
	1.50	23.8	22.8	1.46	56.1	43.6	52.9	0.94
	1.75	32.3	24.4	1.40	47.5	39.5	48.5	0.83
Pooled SEM		4.52	2.49	0.28	3.68	2.89	3.48	0.28
Particle size mean								
Fine		25.9	24.3	1.46	45.2	40.7	47.5	1.25
Coarse		27.8	28.8	1.34	48.6	39.8	50.7	1.60
Pooled SEM		1.64	1.02	0.11	1.50	1.18	1.42	0.11
Ca/P ratio mean								
	0.50	18.1	30.1	1.53	46.3	39.0	48.5	2.37
	0.75	25.5	29.5	1.65	46.5	38.4	51.2	1.96
	1.00	29.7	28.6	1.22	45.3	39.1	48.9	1.57
	1.25	28.0	24.6	1.33	48.3	42.4	46.0	1.05
	1.50	27.2	22.5	1.34	49.7	42.8	51.7	0.88
	1.75	32.6	24.1	1.33	45.6	39.9	48.3	0.72
Pooled SEM		2.76	1.76	0.20	2.60	2.04	2.46	0.19
*P*-value								
Particle size		0.441	0.004	0.441	0.113	0.564	0.618	0.034
Ca/P ratio		0.042	0.014	0.682	0.824	0.550	0.116	<0.001
Particle size × Ca/P ratio		0.410	0.141	0.266	0.343	0.484	0.439	0.512
Linear (Ca/P ratio)		0.004	0.001	0.281	0.684	0.244	0.899	<0.001
Quadratic (Ca/P ratio)		0.271	0.682	0.551	0.641	0.501	0.942	0.240

1 Data are presented as treatment means, 4 replicate pens per treatment (n = 4).

2 MRT = mean retention time; Prov. + gizzard = proventriculus plus gizzard; Prox. ileum = proximal ileum, the first half of ileum.

3 The Ca content and particle distribution of the coarse and fine limestone are shown in Table 1.

## Discussion

The pelleting process was found to retain the dietary particle size treatment imposed by limestone addition. In the feed ash of the coarse limestone diets, 76% of the added particles could be recovered. Full recovery is unlikely in this respect because of an expected (limited) negative effect of pelleting or incineration on limestone particle size. In addition, the GMD of both the coarse and fine limestone diets linearly increase with incremental Ca/P ratio. The increase for the coarse limestone diets was close to the theoretical increase where the slope for the analyzed was 16 vs. 15 for the theoretical linear regression line. In addition, it should be noted that the treatments aimed to impact the Ca and P digestion by affecting the solubility and/or interaction of Ca and P but not gizzard development or surface area available to the digestive enzymes. As such, masking of the limestone particle size difference, given that the diets had less than 2% limestone, by the particles in the diet is integral to the design of the study reported here to only test the effect of limestone particle size and not overall diet particle size. However, it can be ruled out that the observed effects are only related to solubility and/or interaction of Ca and P.

The hypothesis that coarse limestone was more digestible to the broilers was not proven in the present study. The jejunal apparent Ca digestibility was higher for fine than coarse limestone (46.2 vs. 40.8%), but the apparent distal ileal Ca digestibility was similar for coarse and fine limestone (51.5 vs. 49.2%). Therefore, the experimental results did not support the possibility to reduce the Ca/P ratio while maintaining the digestible Ca supply through the use of coarse rather than fine limestone. Compared with fine limestone, coarse limestone slightly improved the distal ileal P digestibility (60.5 and 57.0%). Nevertheless, the coarse limestone could not alleviate the negative effect of a low Ca/P ratio on the CTS or growth performance. As observed by Diaz-Alonso et al. (2019), broilers required more Ca and P to ensure adequate bone mineralization than maximum growth performance. In the current study, both growth performance and CTS were compromised by a low dietary Ca/P ratio, regardless of limestone particle size.

Coarse and fine limestone had a similar Ca digestibility at a low inclusion level in the distal ileum, whereas coarse limestone had a lower Ca digestibility at a high dietary Ca/P ratio. Compared with fine limestone (particle size < 500 μm), coarse limestone (particle size 1,000–2,000 μm) has been reported to have a higher ileal Ca digestibility (70 vs. 40%; Anwar et al., 2016, 2017). The latter authors used coarser limestone compared with the present study (1,000–2,000 vs. 500–2,000 μm). Moreover, mash feed was used by Anwar et al. (2016, 2017), whereas pelleted feed was used in the present study. Adaptation time to the diets might be another reason for the discrepancy between Anwar et al. (2016, 2017) and the present study (3 vs. 6 D, respectively). David et al. (2019) reported that observed distal ileal Ca digestibility decreased linearly with increasing dietary adaptation time, with higher digestibility obtained at 1-D adaptation than 3 or 5 D of adaptation (limestone particle size 370 μm). Limestone transiently accumulated in the crop and gizzard, with a greater concentration effect for coarse limestone (Supplementary Table 3); hence, a longer adaption time might be required for coarse than fine limestone to achieve a steady passage rate of digesta. Owing to the adaptation of broilers to the changes of diets, short-term experiments may not represent the response of broilers over a longer period. In a 6-wk study in broiler breeder hens (Manangi et al., 2018), the large-particle-size limestone decreased the P but not the Ca content in the excreta (average particle size was 185 and 3,490 μm for fine and coarse limestone, respectively). These results agree with our finding, indicating that particle size of limestone probably had an impact on P digestion, whereas it had less impact on Ca digestion. In an in vitro study to mimic the Ca solubilization in the GIT (Kim et al., 2019), fine limestone initially released more Ca than coarse limestone; however, the same amount of Ca was released after 20 min (particle size < 75 and 402 μm for the fine and course limestone, respectively). This in vitro solubility finding is in line with the observed Ca digestibility in the present study, that is the Ca digestibility was higher for the fine limestone in the jejunum, and this difference disappeared in the ileum for the low Ca/P ratios but retained for the high Ca/P ratios. Mechanism behind this interactive effect might be that limestone provided only approximately 50% of Ca in the lowest and close to 90% of the Ca in the highest Ca/P ratio diet. In addition, Ca absorption in the proximal small intestine (jejunum) was not adequate for the low Ca/P ratios, and a substantial amount of Ca was absorbed in the distal small intestine (ileum) for the low Ca/P ratios, irrespective of limestone particle size. The superiority of fine limestone over coarse limestone, therefore, disappeared in the ileum for the low Ca/P ratios, whereas it remained for the high Ca/P ratios. In conclusion, coarse limestone has a limited effect on the distal ileal Ca apparent digestibility at a low Ca/P ratio but may decrease Ca apparent digestibility at a higher Ca/P ratio.

In contrast to Ca digestibility, distal ileal P apparent digestibility was slightly improved by coarse limestone. As mentioned before, the fine limestone released more Ca and improved the Ca apparent digestibility in the upper GIT (jejunum). The rapid Ca release probably promotes insoluble Ca–P complexation in the foregut whereas phytate is primarily hydrolyzed in the upper GIT, specifically in the crop, proventriculus, and gizzard (Dersjant-Li et al., 2015). The relatively rapid Ca release and Ca–P complexation, therefore, may decrease P digestion. In an in vitro study, compared with the limestone with larger particle sizes (137–1,306 μm), fine limestone (28 μm) had a higher Ca solubility and a greater depression on phytase efficacy (Manangi and Coon, 2007). In addition, expression of the P transporters and sodium-dependent phosphate transporter type IIb gradually decreases along the small intestine (Liu et al., 2016; Hu et al., 2018; Shao et al., 2018). Therefore, a rapid Ca release in the upper gut may also decrease P absorption.

The interaction between Ca, P, phytate, and phytase in the gut is complex. Complexation of Ca-P is widely acknowledged to be responsible for Ca depressing P digestion. However, P solubility was not affected by the dietary treatments in the present study. Therefore, P solubility or Ca–P complexation did not seem to be a major factor in the Ca and P interaction. It should be noted that the freeze-dried digesta was used in the present study to conduct the P solubility test, whereas it is unknown if freeze-drying affects the mineral solubility. In an in vitro model (Walk et al., 2012), limestone addition improved P solubility in the gastric phase, whereas it reduced P solubility in the small intestine phase. The mechanism behind Ca stimulating P solubilization is not clear yet, but it implies that the interaction between Ca, phytase, phytate, inorganic P, and phytate-P in the GIT is complicated, and the Ca-P complexation maybe only part of the reason for Ca depressing P digestion.

The effects of dietary Ca/P ratio on growth performance, digesta pH, Ca, and P digestion as well as bone development has been extensively studied (Akter et al., 2016; Gautier et al., 2017; Li et al., 2018). These reports supported our findings that the high dietary Ca/P ratio decreased the ileal Ca and P digestibility. The optimal dietary Ca/P ratio was 1.00–1.25 to maximize Ca and P digestion while maintaining growth performance and CTS. By reducing the Ca/P ratio from 1.75 to 1.00, the distal ileal Ca and P apparent digestibility could be improved from 36.6 to 53.7% and 48.0 to 58.3%, respectively. Reducing dietary Ca/P ratio to below 1.00 impaired CTS, although the Ca and P apparent digestibility could be further improved.

In conclusion, distal ileal Ca apparent digestibility of coarse limestone was equal to fine limestone at a low Ca/P ratio, but it was lower for coarse than fine limestone at a high dietary Ca/P ratio. Coarse limestone slightly improved the distal ileal P apparent digestibility compared with fine limestone. In diets with a total P content of 5.5 g/kg, a reduction of Ca/P ratio improved the Ca and P apparent digestibility, with the optimal Ca/P ratio being 1.00 to 1.25 to optimize the Ca and P digestion while maintaining growth performance and CTS.

## References

[bib1] Akter M., Graham H., Iji P. (2016). Response of broiler chickens to different levels of calcium, non-phytate phosphorus and phytase. Br. Poult. Sci..

[bib2] Anwar M.N., Ravindran V., Morel P.C., Ravindran G., Cowieson A.J. (2016). Effect of limestone particle size and calcium to non-phytate phosphorus ratio on true ileal calcium digestibility of limestone for broiler chickens. Br. Poult. Sci..

[bib3] Anwar M., Ravindran V., Morel P., Ravindran G., Cowieson A. (2017). Effect of calcium source and particle size on the true ileal digestibility and total tract retention of calcium in broiler chickens. Anim. Feed Sci. Technol..

[bib4] ASAE (2008). Method of Determining and Expressing Fineness of Feed Materials by Sieving. American Society of Agricultural Engineers Standard S319.4.

[bib5] CVB (2016). Feeding Standards and Nutritional Values of Feeding Ingredients for Poultry.

[bib6] David L.S., Abdollahi M.R., Ravindran G., Walk C.L., Ravindran V. (2019). Studies on the measurement of ileal calcium digestibility of calcium sources in broiler chickens. Poult. Sci..

[bib7] de Vries S., Gerrits W.J., Moughan P.J., Hendriks W.H. (2018). The use of tracers or markers in digestion studies. Feed Evaluation Science.

[bib8] Dersjant-Li Y., Awati A., Schulze H., Partridge G. (2015). Phytase in non-ruminant animal nutrition: a critical review on phytase activities in the gastrointestinal tract and influencing factors. J. Sci. Food Agric..

[bib9] Díaz-Alonso J.A., Gómez-Rosales S., Angeles M.d.L., Ávila-González E., López-Coello C. (2019). Effects of the level and relationship of calcium and available phosphorus on the growth and tibia mineralization of broiler starter chickens. J. Appl. Poult. Res..

[bib10] Gautier A.E., Walk C.L., Dilger R.N. (2017). Influence of dietary calcium concentrations and the calcium-to-non-phytate phosphorus ratio on growth performance, bone characteristics, and digestibility in broilers. Poult. Sci..

[bib11] Guz B.C., Molenaar R., de Jong I.C., Kemp B., van den Brand H., van Krimpen M. (2019). Effects of dietary organic minerals, fish oil, and hydrolyzed collagen on growth performance and tibia characteristics of broiler chickens. Poult. Sci..

[bib12] Hu Y., Liao X., Wen Q., Lu L., Zhang L., Luo X. (2018). Phosphorus absorption and gene expression levels of related transporters in the small intestine of broilers. Br. J. Nutr..

[bib13] ISO (1998). 6491: Animal Feeding Stuffs - Determination of Phosphorus Content - Spectrometric Method.

[bib14] ISO (1999). 6496: Animal Feeding Stuffs - Determination of Moisture and Other Volatile Matter Content.

[bib15] ISO (2000). 6869: Animal Feeding Stuffs - Determination of the Contents of Calcium, Copper, Iron, Magnesium, Manganese, Potassium, Sodium and Zinc - Method Using Atomic Absorption Spectrometry.

[bib16] ISO (2002). 5984: Animal Feeding Stuffs - Determination of Crude Ash.

[bib17] ISO (2005). 5983-1: Animal Feeding Stuffs - Determination of Nitrogen Content and Calculation of Crude Protein Content - Part 1: Kjeldahl Method.

[bib18] Kim S.W., Li W., Angel R., Proszkowiec-Weglarz M. (2018). Effects of limestone particle size and dietary Ca concentration on apparent P and Ca digestibility in the presence or absence of phytase. Poul. Sci..

[bib19] Kim S.W., Li W., Angel R., Plumstead P.W. (2019). Modification of a limestone solubility method and potential to correlate with in vivo limestone calcium digestibility. Poult. Sci..

[bib20] Li W., Angel R., Kim S.W., Jiménez-Moreno E., Proszkowiec-Weglarz M., Plumstead P.W. (2018). Impacts of age and calcium on phytase efficacy in broiler chickens. Anim. Feed Sci. Technol..

[bib21] Liu S.B., Hu Y.X., Liao X.D., Lu L., Li S.F., Zhang L.Y., Tan H.Z., Yang L., Suo H.Q., Luo X.G. (2016). Kinetics of phosphorus absorption in ligated small intestinal segments of broilers. J. Anim. Sci..

[bib22] Manangi M., Coon C. (2007). The effect of calcium carbonate particle size and solubility on the utilization of phosphorus from phytase for broilers. Int. J. Poult. Sci..

[bib23] Manangi M.K., Maharjan P., Coon C.N. (2018). Calcium particle size effects on plasma, excreta, and urinary Ca and P changes in broiler breeder hens. Poult. Sci..

[bib24] Misiura M.M., Filipe J.A.N., Walk C.L., Kyriazakis I. (2018). Do not neglect calcium: a systematic review and meta-analysis (meta-regression) of its digestibility and utilisation in growing and finishing pigs. Br. J. Nutr..

[bib25] Myers W., Ludden P., Nayigihugu V., Hess B. (2004). A procedure for the preparation and quantitative analysis of samples for titanium dioxide. J. Anim. Sci..

[bib26] Rodehutscord M. (2013). Determination of phosphorus availability in poultry. World’s Poult. Sci. J..

[bib27] Rodehutscord M., Walk C.L., Kühn I., Stein H.H., Kidd M.T., Rodehutscord M. (2016). Chapter 10 Interactions between minerals and phytate degradation in poultry–challenges for phosphorus digestibility assays. Phytate Destruction-Consequences for Precision Animal Nutrition.

[bib28] ROSS (2014). Ross 308 Broiler: Performance Objective. http://en.aviagen.com/assets/Tech_Center/Ross_Broiler/Ross-308-Broiler-PO-2014-EN.pdf.

[bib29] Shao Y., Sun G., Cao S., Lu L., Zhang L., Liao X., Luo X. (2019). Bone phosphorus retention and bone development of broilers at different ages. Poult. Sci..

[bib30] Shao Y., Wen Q., Zhang S., Lu L., Zhang L., Liao X., Luo X. (2018). Dietary supplemental vitamin D_3_ enhances phosphorus absorption and utilisation by regulating gene expression of related phosphate transporters in the small intestine of broilers. Br. J. Nutr..

[bib31] Van Krimpen M.M., van Diepen J.T.M., van Wikselaar P., Bikker P., Jongbloed A.W. (2013). Effects of Available Phosphorus (aP), calcium/aP Ratio, and Growth Rate on P Deposition, P Digestibility, Performance and Leg Quality in Broilers.

[bib32] Walk C.L., Bedford M.R., McElroy A.P. (2012). *In vitro* evaluation of limestone, dicalcium phosphate, and phytase on calcium and phosphorus solubility of corn and soybean meal. Poult. Sci..

