# Supplementary material for: Coarse limestone does not alleviate the negative effect of a low Ca/P ratio diet on characteristics of tibia strength and growth performance in broilers
Source: Poult Sci. 2020 Jul 3;99(10):4978–89. doi: 10.1016/j.psj.2020.06.037 (PMC7598140; doi:10.1016/j.psj.2020.06.037)
Supplement: Supplementary Tables 1–3 [file mmc1.docx]

| **Supplementary Table 1**. Composition and nutrient content of finisher diets with fine or coarse limestone and incremental Ca/P ratios^1^ (g/kg, as-fed basis, d 30-39) | | | | | | | | | | | | | |
| --- | --- | --- | --- | --- | --- | --- | --- | --- | --- | --- | --- | --- | --- |
| Item | Particle size limestone | | | | | | | | | | | | |
|  | Fine | | | | | |  | Coarse | | | | | |
| Ca/P ratio | 0.50 | 0.75 | 1.00 | 1.25 | 1.50 | 1.75 |  | 0.50 | 0.75 | 1.00 | 1.25 | 1.50 | 1.75 |
| Ingredients |  |  |  |  |  |  |  |  |  |  |  |  |  |
| Corn | 363 | 357 | 351 | 345 | 339 | 333 |  | 363 | 357 | 351 | 345 | 339 | 333 |
| Wheat | 350 | 350 | 350 | 350 | 350 | 350 |  | 350 | 350 | 350 | 350 | 350 | 350 |
| Soybean meal, extracted | 238 | 238 | 239 | 239 | 240 | 240 |  | 238 | 238 | 239 | 239 | 240 | 240 |
| Soybean oil | 26 | 28 | 30 | 32 | 34 | 37 |  | 26 | 28 | 30 | 32 | 34 | 37 |
| Monosodium phosphate | 6.1 | 6.1 | 6.1 | 6.1 | 6.1 | 6.1 |  | 6.1 | 6.1 | 6.1 | 6.1 | 6.1 | 6.1 |
| Monocalcium phosphate | 2.0 | 2.0 | 2.0 | 2.0 | 2.0 | 2.0 |  | 2.0 | 2.0 | 2.0 | 2.0 | 2.0 | 2.0 |
| Limestone (fine) | 3.5 | 6.8 | 10.2 | 13.5 | 16.8 | 20.0 |  | 0 | 0 | 0 | 0 | 0 | 0 |
| Limestone (coarse) | 0 | 0 | 0 | 0 | 0 | 0 |  | 3.5 | 6.8 | 10.2 | 13.5 | 16.8 | 20.0 |
| Salt | 0.2 | 0.2 | 0.2 | 0.2 | 0.2 | 0.2 |  | 0.2 | 0.2 | 0.2 | 0.2 | 0.2 | 0.2 |
| L-Val (98%) | 0.7 | 0.7 | 0.7 | 0.7 | 0.7 | 0.7 |  | 0.7 | 0.7 | 0.7 | 0.7 | 0.7 | 0.7 |
| Met (99%) | 2.3 | 2.3 | 2.3 | 2.3 | 2.3 | 2.3 |  | 2.3 | 2.3 | 2.3 | 2.3 | 2.3 | 2.3 |
| L-Lys (79%) | 2.8 | 2.8 | 2.8 | 2.8 | 2.8 | 2.8 |  | 2.8 | 2.8 | 2.8 | 2.8 | 2.8 | 2.8 |
| Thr (98%) | 1.0 | 1.0 | 1.0 | 1.0 | 1.0 | 1.0 |  | 1.0 | 1.0 | 1.0 | 1.0 | 1.0 | 1.0 |
| Titanium dioxide (TiO_2_) | 5.0 | 5.0 | 5.0 | 5.0 | 5.0 | 5.0 |  | 5.0 | 5.0 | 5.0 | 5.0 | 5.0 | 5.0 |
| Premix^2^ | 5.0 | 5.0 | 5.0 | 5.0 | 5.0 | 5.0 |  | 5.0 | 5.0 | 5.0 | 5.0 | 5.0 | 5.0 |
| Calculated nutrients |  |  |  |  |  |  |  |  |  |  |  |  |  |
| Dry matter | 875 | 876 | 876 | 877 | 878 | 878 |  | 875 | 876 | 876 | 877 | 878 | 878 |
| ME, kcal/kg | 3059 | 3059 | 3059 | 3059 | 3059 | 3059 |  | 3059 | 3059 | 3059 | 3059 | 3059 | 3059 |
| Crude protein^3^ | 188 | 188 | 188 | 187 | 187 | 187 |  | 188 | 188 | 188 | 187 | 187 | 187 |
| Lys | 10 | 10 | 10 | 10 | 10 | 10 |  | 10 | 10 | 10 | 10 | 10 | 10 |
| Met | 5.0 | 5.0 | 5.0 | 5.0 | 5.0 | 5.0 |  | 5.0 | 5.0 | 5.0 | 5.0 | 5.0 | 5.0 |
| Met+Cys | 8.2 | 8.2 | 8.2 | 8.2 | 8.2 | 8.2 |  | 8.2 | 8.2 | 8.2 | 8.2 | 8.2 | 8.2 |
| Thr | 7.6 | 7.6 | 7.6 | 7.6 | 7.6 | 7.6 |  | 7.6 | 7.6 | 7.6 | 7.6 | 7.6 | 7.6 |
| Ca | 2.5 | 3.8 | 5.1 | 6.3 | 7.6 | 8.8 |  | 2.5 | 3.8 | 5.1 | 6.3 | 7.6 | 8.8 |
| Total P (P) | 5.1 | 5.1 | 5.1 | 5.1 | 5.0 | 5.0 |  | 5.1 | 5.1 | 5.1 | 5.1 | 5.0 | 5.0 |
| Available P (aP) | 2.9 | 2.9 | 2.9 | 2.9 | 2.9 | 2.9 |  | 2.9 | 2.9 | 2.9 | 2.9 | 2.9 | 2.9 |
| Ca/P | 0.50 | 0.75 | 1.00 | 1.25 | 1.50 | 1.75 |  | 0.50 | 0.75 | 1.00 | 1.25 | 1.50 | 1.75 |
| Analysed nutrients |  |  |  |  |  |  |  |  |  |  |  |  |  |
| Dry matter | 877 | 882 | 884 | 884 | 884 | 884 |  | 883 | 885 | 885 | 885 | 884 | 883 |
| Crude protein | 184 | 182 | 182 | 183 | 183 | 182 |  | 182 | 181 | 185 | 184 | 185 | 181 |
| Crude fat | 57.8 | 58.8 | 57.3 | 59.3 | 59.2 | 58.4 |  | 58.9 | 59.2 | 59.8 | 56.1 | 55.6 | 56.2 |
| Ca | 2.8 | 4.1 | 5.4 | 6.7 | 7.7 | 9.0 |  | 3.0 | 3.7 | 5.3 | 6.5 | 7.2 | 8.2 |
| P | 5.3 | 5.5 | 5.8 | 5.7 | 5.6 | 5.7 |  | 5.7 | 5.7 | 5.7 | 5.6 | 5.8 | 5.3 |
| Ca/P | 0.53 | 0.75 | 0.93 | 1.18 | 1.38 | 1.58 |  | 0.53 | 0.65 | 0.93 | 1.16 | 1.24 | 1.55 |
| ^1^ The Ca content and particle size distribution of the coarse and fine limestone are shown in Table 1.  ^2^ Provided per kg of diet: 12,000 IE retinol, 2,400 IE cholecalciferol, 50 mg dl-a-tocopherol, 1.5 mg menadione, 2.0 mg thiamine, 7.5 mg riboflavin, 3.5 mg pyridoxine, 20 mg cyanocobalamins, 35 mg niacin, 12 mg D-pantothenic acid, 460 mg choline chloride, 1.0 mg folic acid, 0.2 mg biotin, 80 mg iron, 12 mg copper, 85 mg manganese, 60 mg zinc, 0.4 mg cobalt, 0.8 mg iodine, 0.1 mg selenium, 125 mg anti-oxidant mixture. | | | | | | | | | | | | | |

| **Supplementary Table 2**. Effect of Ca/P ratio and particle size of limestone on growth performance in the finisher and overall broilers^1,2,3,4^ | | | | | | | | | | | | | | | |
| --- | --- | --- | --- | --- | --- | --- | --- | --- | --- | --- | --- | --- | --- | --- | --- |
| Particle size | Ca/P ratio |  | BW, g | | | |  | Finisher period (d 29-39) | | |  | Overall period (d 14-39) | | |  |
|  |  |  | d 14 | d 20 | d 29 | d 39 |  | BWG,  g | FI,  g | FCR,  g/g |  | BWG,  g | FI,  g | FCR,  g/g |  |
| Fine | 0.50 |  | 431 | 893 | 1702 | 2756 |  | 1054 | 1918 | 1.83 |  | 2325 | 3630 | 1.56 |  |
|  | 0.75 |  | 436 | 918 | 1866 | 3034 |  | 1168 | 2113 | 1.83 |  | 2597 | 4035 | 1.56 |  |
|  | 1.00 |  | 425 | 907 | 1822 | 3035 |  | 1213 | 2091 | 1.73 |  | 2609 | 3888 | 1.49 |  |
|  | 1.25 |  | 430 | 915 | 1874 | 3043 |  | 1169 | 2137 | 1.84 |  | 2613 | 4018 | 1.54 |  |
|  | 1.50 |  | 431 | 927 | 1921 | 3007 |  | 1086 | 2082 | 1.97 |  | 2576 | 3957 | 1.55 |  |
|  | 1.75 |  | 428 | 885 | 1811 | 2897 |  | 1086 | 2001 | 1.87 |  | 2469 | 3804 | 1.54 |  |
| Coarse | 0.50 |  | 428 | 872 | 1665 | 2705 |  | 1040 | 1907 | 1.86 |  | 2277 | 3620 | 1.59 |  |
|  | 0.75 |  | 431 | 909 | 1813 | 2957 |  | 1145 | 2029 | 1.78 |  | 2526 | 3885 | 1.54 |  |
|  | 1.00 |  | 432 | 922 | 1831 | 2987 |  | 1156 | 2030 | 1.78 |  | 2555 | 3868 | 1.52 |  |
|  | 1.25 |  | 430 | 919 | 1844 | 2951 |  | 1107 | 2034 | 1.87 |  | 2521 | 3872 | 1.54 |  |
|  | 1.50 |  | 439 | 926 | 1823 | 3006 |  | 1182 | 2060 | 1.75 |  | 2566 | 3853 | 1.50 |  |
|  | 1.75 |  | 435 | 928 | 1906 | 3093 |  | 1187 | 2167 | 1.85 |  | 2659 | 4055 | 1.53 |  |
| Pooled SEM | |  | 5.07 | 10.7 | 40.6 | 68.8 |  | 57.7 | 51.7 | 0.09 |  | 67.6 | 120 | 0.05 |  |
| Particle size mean | |  |  |  |  |  |  |  |  |  |  |  |  |  |  |
| Fine | |  |  | 907 | 1833 | 2958 |  | 1128 | 2055 | 1.84 |  | 2532 | 3889 | 1.54 |  |
| Coarse | |  |  | 910 | 1814 | 2950 |  | 1136 | 2038 | 1.82 |  | 2517 | 3859 | 1.54 |  |
| Pooled SEM | |  |  | 4.4 | 16.6 | 28.1 |  | 23.6 | 21.1 | 0.04 |  | 27.6 | 49.1 | 0.02 |  |
| Ca/P ratio mean | |  |  |  |  |  |  |  |  |  |  |  |  |  |  |
|  | 0.50 |  |  | 883 | 1684 | 2731 |  | 1047 | 1913 | 1.84 |  | 2301 | 3625 | 1.58 |  |
|  | 0.75 |  |  | 914 | 1840 | 2996 |  | 1156 | 2071 | 1.81 |  | 2562 | 3960 | 1.55 |  |
|  | 1.00 |  |  | 915 | 1827 | 3011 |  | 1182 | 2058 | 1.75 |  | 2582 | 3878 | 1.51 |  |
|  | 1.25 |  |  | 917 | 1859 | 2997 |  | 1138 | 2085 | 1.86 |  | 2567 | 3945 | 1.54 |  |
|  | 1.50 |  |  | 927 | 1872 | 3007 |  | 1139 | 2070 | 1.85 |  | 2571 | 3905 | 1.53 |  |
|  | 1.75 |  |  | 906 | 1859 | 2995 |  | 1137 | 2084 | 1.86 |  | 2564 | 3930 | 1.54 |  |
| Pooled SEM | |  |  | 7.5 | 28.7 | 48.6 |  | 40.8 | 36.5 | 0.07 |  | 47.8 | 85.0 | 0.04 |  |
| *P*-value |  |  | 0.819 |  |  |  |  |  |  |  |  |  |  |  |  |
| Particle size |  |  |  | 0.423 | 0.431 | 0.768 |  | 0.839 | 0.528 | 0.601 |  | 0.746 | 0.676 | 0.897 |  |
| Ca/P ratio |  |  |  | 0.004 | <0.001 | 0.001 |  | 0.303 | 0.011 | 0.845 |  | 0.001 | 0.070 | 0.807 |  |
| Particle size × Ca/P ratio | | |  | 0.076 | 0.290 | 0.321 |  | 0.570 | 0.135 | 0.755 |  | 0.337 | 0.571 | 0.970 |  |
| Linear (Ca/P ratio) | |  |  | 0.015 | <0.001 | 0.002 |  | 0.333 | 0.006 | 0.530 |  | 0.002 | 0.051 | 0.465 |  |
| Quadratic (Ca/P ratio) | |  |  | 0.002 | 0.007 | 0.003 |  | 0.086 | 0.032 | 0.486 |  | 0.003 | 0.084 | 0.315 |  |
| ^1^ Data are presented as treatment means, 6 replicate pens per treatment (n=6).  ^2^ BW=body weight; BWG=body weight gain; FI=feed intake; FCR=feed conversion ratio.  ^3^ The Ca content and particle distribution of the coarse and fine limestone are shown in Table 1.  ^4^ Each treatment had an equal number of 6 pens with 16 birds per pen before d 20, and an unequal number of 4 pens with 4 birds per pen and 2 pens with 16 birds per pen after d 21. | | | | | | | | | | | | | | | |

| **Supplementary Table 3**. Effect of Ca/P ratio and particle size of limestone on Ca and P apparent digestibility in crop, gizzard, duodenum and ceca in broilers^1,2,3^, % | | | | | | | | | | |
| --- | --- | --- | --- | --- | --- | --- | --- | --- | --- | --- |
| Particle size | Ca/P ratio | P disappearance/digestibility | | | |  | Ca disappearance/digestibility | | | |
|  |  | Crop | Prov.+Gizzard | Duodenum | Ceca |  | Crop | Prov.+Gizzard | Duodenum | Ceca |
| Fine | 0.50 | 6.07 | 53.2 | -73.2 | -264 |  | -7.1 | 36.1 | -84.9 | -111 |
|  | 0.75 | 1.13 | 51.1 | -66.3 | -284 |  | -5.7 | 33.3 | -75.6 | -184 |
|  | 1.00 | 3.50 | 52.4 | -90.9 | -348 |  | -1.2 | 40.8 | -74.4 | -247 |
|  | 1.25 | 2.50 | 43.8 | -74.9 | -528 |  | -1.8 | -21.7 | -131 | -698 |
|  | 1.50 | 1.21 | 44.4 | -39.1 | -654 |  | -15.2 | -81.4 | -195 | -1057 |
|  | 1.75 | -2.00 | 46.5 | -93.4 | -811 |  | -23.6 | -13.7 | -77.3 | -1334 |
| Coarse | 0.50 | 6.57 | 56.6 | -76.0 | -191 |  | -9.9 | -42.1 | -149 | -56.9 |
|  | 0.75 | 9.02 | 56.9 | -43.9 | -259 |  | -7.2 | -73.5 | -442 | -112 |
|  | 1.00 | 3.80 | 50.1 | -51.6 | -309 |  | -4.1 | -88.2 | -284 | -193 |
|  | 1.25 | 0.30 | 51.2 | -91.9 | -484 |  | -17.2 | -64.1 | -175 | -496 |
|  | 1.50 | -1.39 | 43.7 | -91.8 | -508 |  | -44.3 | -191 | -190 | -652 |
|  | 1.75 | 0.93 | 43.2 | -84.1 | -699 |  | -35.4 | -392 | -109 | -958 |
| ^1^ Data are presented as treatment means, 4 replicate pens per treatment (n=4).  ^2^ Prov.+gizzard=proventriculus plus gizzard.  ^3^ The Ca content and particle distribution of the coarse and fine limestone are shown in Table 1. | | | | | | | | | | |
